# Application of PS2M Aptamer as Receptor Layer for Electrochemical Detection of Lead Ions

**DOI:** 10.3390/bios15010059

**Published:** 2025-01-17

**Authors:** Izabela Zaras, Olga Kujawa, Marcin Olszewski, Marta Jarczewska

**Affiliations:** 1 Chair of Medical Biotechnology, Faculty of Chemistry, Warsaw University of Technology, Noakowskiego 3, 00-664 Warsaw, Poland; izabela.zaras.dokt@pw.edu.pl (I.Z.);; 2 Chair of Drug and Cosmetics Biotechnology, Faculty of Chemistry, Warsaw University of Technology, Koszykowa 75, 00-664 Warsaw, Poland; marcin.olszewski@pw.edu.pl

**Keywords:** aptamer-sensing layer, voltammetry, redox indicators, modified electrodes

## Abstract

Since lead can cause severe effects on living organisms’ health and life, the regular monitoring of Pb levels in water and soil is of particular significance. Recently, it was shown that lead ions can also be detected using affinity-based biosensors, namely, using aptamers as recognition elements. In most cases, thrombin binding aptamer (TBA) was utilized; however, there are more examples of DNA aptamers which could also serve that purpose. Herein, we present studies on the electrochemical detection of lead ions using PS2M aptamer, which contains several guanine nucleotides, as the receptor element. Firstly, the method of aptamer-based layer fabrication was optimized along with the choice of a redox active indicator, which was a source of current signal. The experiments revealed the possibility of lead ion detection from 50 to 600 nM, which covers the range below and above the maximum accepted limit stated by US EPA (72 nM). Moreover, the sensing layer exhibited high selectivity towards lead ions and was successfully applied both for the analysis of tap water spiked with Pb^2+^ ions and as a miniaturized sensor. Finally, stability and regeneration studies on the aptamer-based receptor layer were executed to confirm the utility of the elaborated tool.

## 1. Introduction

The necessity to regularly control the quality of drinking water is justified by the introduction of contaminations because of the development of various areas such as urbanization, agriculture, and mining. One of the most severe outcomes is the presence of heavy metals in surface and ground waters among which one of the most dangerous is lead. Lead is currently used in the production of pottery, pipes, car batteries, ammunition, or even stained-glass windows [1]. It should be noted that Pb might accumulate in various organs and thus influence the state of the nervous system, the muscles, and the renal system. Lead exhibits affinity towards sulfhydryl groups, among others, and interacts with enzymes such as delta-aminolevulinic acid dehydratase (ALAD) and glutathione reductase (GR). The inhibition of ALAD causes an increase in delta-aminolevulinic acid (ALA) and that is why heme production is decreased [2]. That is one of the reasons why the regular monitoring of lead levels is important, and among the traditionally utilized methods are capillary electrophoresis, atomic absorbance spectrometry [3,4,5], inductively coupled plasma spectrometry, and optical methods [6,7,8,9]. Among the main limitations of the above-mentioned techniques is the need to use sophisticated equipment and trained operators; moreover, the portability of such classical methods is limited, which does not allow for a reduction in the period required for sample analysis. To overcome such challenges, electrochemical methods including potentiometry [10] combined with ion-selective electrodes (ISEs) [11,12], as well as voltammetry including stripping methods [13,14,15,16,17,18,19,20,21], are widely applied.

Metal ions can also be detected using biosensors containing receptor layers formed of nucleic acids. The application of solely single-stranded DNA might not provide the actual selectivity of recognition layer especially when a complex sample is investigated. This might be solved using “functional nucleic acids”, which are defined as DNA or RNA sequences exhibiting both receptor and catalytic features that can also interact with certain metal cations such as aptamers, DNAzymes, and allosteric nucleic acids [22]. For lead ion detection, the use of both DNAzymes [23,24,25,26,27] and aptamers [28,29,30,31,32,33,34,35,36] was investigated. The latter are known as single-stranded DNA or RNA sequences which change their conformation upon interaction with target analytes of diverse molecular weight, starting from metal cations [37], through proteins and cells. Aptamers are obtained through the systematic evolution of ligands by exponential enrichment (SELEX) process that enables the choice of a limited group of aptamer probes from large libraries of DNA or RNA sequences [38]. Aptamers can be successfully applied to the fabrication of receptor layers thanks to the possibility of introducing groups enabling surface functionalization or labels allowing for optical or electrochemical detection. Aptamer receptor layers are beneficial not only because of their high binding affinities that are comparable with those of antibody-based layers, but also because they are reversible and resistant to harsh temperature and pH conditions. The determination of lead ions using aptamer strands is possible as this metal cation is capable of causing a conformation transition of single-stranded nucleic acid sequences abundant with guanine moieties. Such interaction results in the formation of a G-quadruplex structure which is known as non-canonical DNA secondary construct and is composed of planar stacks of four guanines stabilized by Hoogsteen hydrogen bonding [39,40,41].

Lead ions were successfully detected using guanine-rich sequences, which was mostly realized through the application of a thrombin binding aptamer (TBA). Upon interaction between the G-rich aptamer and Pb^2+^ ions, a G-quadruplex structure was formed that caused a rearrangement of the sensing layer and the exposure of guanine moieties to the outer part of the sensing layer. This phenomenon was the basis for the operation of aptasensors elaborated by our group [42,43], which additionally employed an external redox indicator, methylene blue, which was a source of current response. The proposed aptamer-based biosensors allowed for lead ion detection below the maximum acceptable level [42,43] and exhibited high selectivity towards target analytes.

**Scheme 1:**
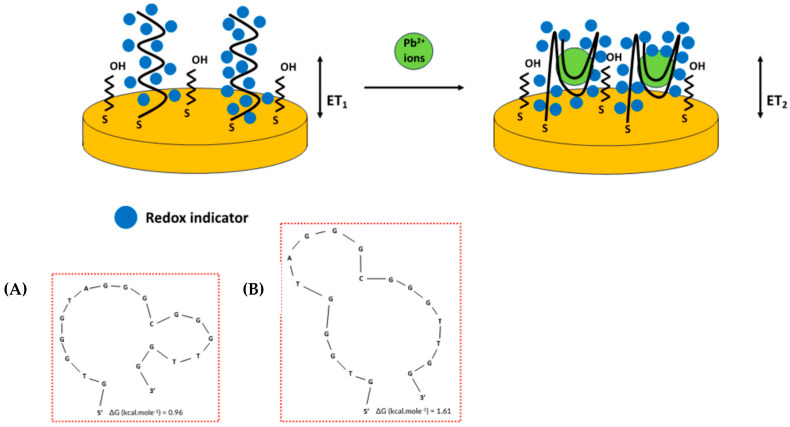
Schematic representation of aptamer-based layer behavior and formation of G-quadruplex structure after incubation with lead ions along with two most favorable PS2M aptamer conformations (**A**,**B**) in the absence of Pb^2+^ ions.

In the present study, we aimed to utilize another G-rich sequence, namely, an 18-nucleotide aptamer known as PS2M [44], which had not yet been applied to the electrochemical detection of lead ions and was expected to behave similarly to the TBA probe and form a G-quadruplex structure upon interaction with Pb^2+^ ions. PS2M sequences was also shown to bind efficiently with potassium ions [45] and iron (III)-protoporphyrin IX (hemin) [46]. The guanine tetrads may be composed of a single aptamer sequence; however, constructs made of two or even four separate strands are also possible. The initial conformation of the guanine-rich sequence of PS2M does not form a G-quadruplex structure in the absence of lead ions, as shown in Scheme 1, but rather a hairpin-loop-based structure evidenced by the two conformations exhibiting the most favorable Gibbs energy values (Scheme 1A,B). However, upon the introduction of Pb^2+^ ions, a significant change in aptamer-based layer orientation on the transducer surface should occur. To evidence the changes in the properties of the aptamer-based receptor layer, the initial studies involved the choice of an external electroactive indicator that was the source of electrochemical response as well as conditions for the fabrication of the receptor layer. The optimized conditions enabled the definition of the working parameters of the aptasensor such as the linear range of response, selectivity, and feasibility of use for the analysis of real samples. Finally, regeneration and stability studies were conducted to further verify the benefits of the application of such an aptasensing platform.

## 2. Materials and Methods

### 2.1. Apparatus

Electrochemical measurements were conducted using cyclic voltammetry (CV) and square-wave voltammetry (SWV) with the application of a CHI 660A potentiostat (CH Instruments, Bee Cave, TX, USA). The experiments were performed using a classical three-electrode system consisting of a gold disk electrode (CH Instruments, Bee Cave, TX, USA), an Ag/AgCl/1.0 mol·L^−1^ KCl reference electrode (Mineral, Warsaw, Poland), and a gold wire as an auxiliary electrode (Sigma Aldrich, Darmstadt, Germany) at room temperature. Cyclic voltammetry was conducted at a scan rate of 0.1 V·s^−1^ and square-wave voltammetry at a pulse amplitude of 10 mV, an increment of 5 mV, and frequencies of 15 Hz. The applied potential range varied depending on the type of redox active indicator and the following values were used: −0.2 to 0.6 V for ferri/ferrocyanide (Fe(CN)_6_^3−/4−^), −0.6 to 0.2 V for methylene blue, −0.8 to −0.2 V for anthraquinone-2-sodium methylsulphonate (AQMS), and −0.4 to 0 V for ruthenium(III) hexaammine (RuHex). Part of the stability studies was conducted by placing aptamer-modified electrodes in the climate chamber ICH110L (Memmert GmbH, Schwabach, Germany) for a defined time with the application of selected temperature values and humidity conditions.

### 2.2. Chemicals

Tris-HCl, potassium hexacyanoferrate (III) (K_3_Fe(CN)_6_), potassium hexacyanoferrate (II) (K_4_Fe(CN)_6_), methylene blue (MB), manganese (II) nitrate hydrate, uranyl acetate, lead nitrate, cadmium nitrate, mercury chloride, sodium selenate, nickel sulfate, potassium antimony (III) tartrate hydrate, arsenic (III) ICP standard, 6-mercapto-1-hexanol (MCH), potassium dihydrogen phosphate, bovine serum albumin (BSA), glucose, nitrilotriacetic acid (NTA), and ethylenediaminetetraacetic acid (EDTA) were purchased from Aldrich Chemicals, Germany. Sulfuric acid, potassium chloride, potassium chromate, and hydrogen peroxide were purchased from Avantor Performance Materials Poland S.A., Gliwice, Poland.

DNA aptamer (desalted, purified by HPLC), known as PS2M aptamer, specific for lead ions, was purchased from Metabion, Planegg, Germany. The sequence was as follows: 5′-OH-C_6_-S-S-C_6_-GTGGGTAGGGCGGGTTGG-3′. Aptamer stock solutions (100 μM) were prepared with nuclease-free water and stored in a −20 °C freezer before use.

### 2.3. Solutions

The solutions used in electrochemical measurements were as follows: 50 mM Tris-HCl, pH 4.0, 5 mM ferri/ferrocyanide in 50 mM Tris-HCl, 100 µM AQMS in 50 mM Tris-HCl, 1 and 50 µM methylene blue (MB), and 100 µM RuHex in 50 mM Tris-HCl, 5 mM ferri/ferrocyanide in 0.1 M KCl, 0.1 M H_2_SO_4_, piranha solution (H_2_SO_4_: H_2_O_2_ (3:1) (*v*/*v*)), 1 M KH_2_PO_4_, pH 4.5 (aptamer immobilization buffer), 4 μM, 100 μM, 2 mM MCH in 1 M KH_2_PO_4_, 1 mM NTA in 50 mM Tris-HCl or water, and 1 mM EDTA in 50 mM Tris-HCl (pH 8.0). For the optimization of aptasensor performance as well as the determination of the working parameters, the redox indicator solutions were spiked with lead nitrate or other heavy metal salts to obtain a defined concentration.

### 2.4. Gold Electrode Cleaning and Modification

Gold disk electrodes that were used as working electrodes were initially cleaned starting from mechanical polishing on microcloth pads (Buehler, Lake Bluff, IL, USA) using Al_2_O_3_ powders of grain size 0.3 and 0.05 µm. After washing with distilled water, the electrodes were sonicated at room temperature in H_2_O/ethanol solution (1:1, *v*/*v*) for 5 min This was followed by electrode incubation in piranha solution (H_2_SO_4_:H_2_O_2_ (3:1 *v*/*v*)) for 2 min, then washing with distilled water, and electrochemical scanning using cyclic voltammetry in 1 M H_2_SO_4_ at a scan rate of 0.3 V·s^−1^, for 10 cycles. The purity of the gold electrode surface was confirmed based on the cyclic voltammograms obtained in 0.1 M H_2_SO_4_ conducted at a scan rate of 0.3 V·s^−1^, a potential range from −0.3 V to 1.7 V, 2 cycles, as well as in 5 mM ferri/ferrocyanide in 0.1 M KCl with a scan rate of 0.1 V·s^−1^ for a potential range from −0.2 to 0.6 V, 2 cycles. The electrodes were modified immediately after cleaning by 2 h incubation with 4 µM aptamer solution and then washed with immobilization buffer. This was followed by 1 h incubation in MCH blocking agent solution of a defined concentration.

In the miniaturization studies, screen-printed chips containing gold working electrode (BVT Technologies, Palmsens, Houten, The Netherlands) were used; the cleaning concerned solely washing with distilled water and ethanol, followed by gold surface purity using cyclic voltammetry in 5 mM ferri/ferrocyanide in 0.1 M KCl (scan rate 0.1 V·s^−1^, potential range from −0.2 to 0.6 V, 2 cycles). Further surface modification was identical as for gold macroelectrodes.

## 3. Results

The possibility of the application of aptamer strands to lead ion detection has already been shown in terms of usage of mostly thrombin binding aptamer, which consists of 15 nucleotides with a high abundance of guanine moieties. In recent years, several examples of both electrochemical and optical sensors have been presented, including the introduction of nanomaterials as intermediate layers formed on working electrodes. However, there are more possible aptamer strands which could be used for that purpose such as PS2M. PS2M was utilized in our previous research for the electrochemical detection of potassium cations [47], and there have also been some attempts to determine the presence of lead ions using optical aptasensors [48]. Therefore, we aimed to focus on the feasibility of usage of gold disk electrodes modified with PS2M aptamer for lead ion detection. It was expected that the sensing layer formed by such an aptamer, which also contains a high number of guanines, will behave in a similar manner as in the case of a TBA-based receptor layer. As was indicated in [42,43], upon the addition of lead ions, there was a substantial increase in the current response of the TBA-based biosensor. This was caused by the attraction of methylene blue redox indicator molecules to the sensing layer as the outcome of the exposure of guanine moieties to the outer part of the aptamer-based layer. This is justified by a distinct affinity of methylene blue towards guanine [49]. It should also be noted that numerous mono- and divalent cations are engaged in the stabilization of G-quadruplex including the Hardin order, K^+^ > Ca^2+^ > Na^+^ > Mg^2+^ > Li^+^ and K^+^ > Rb^+^ > Cs^+^ [50], and the Venczel order, Sr^2+^ > Ba^2+^ > Ca^2+^ > Mg^2+^ and K^+^ > Rb^+^ >Na^+^ > Li^+^ = Cs^+^ [51]. Concerning lead ions, they are believed to stabilize a G-quadruplex structure in a three-step process [52]. Furthermore, NMR studies and crystal structures of lipophilic guanosine analogs revealed a smaller G8 octamer cage than that observed for potassium ions. This provides a more kinetically stable Pb^2+^-G8 complex than the K^+^-G8 complex and hence the binding between Pb^2+^ and the guanine-rich aptamer is characterized by higher stability than the K^+^-G-rich sequence interaction [53]. The exceptional properties of the aptamer-layer rearrangement in the presence of lead ions were confirmed by impedance spectroscopy experiments, also evidencing the necessity of introducing Pb^2+^ and guanine-rich sequences as the receptor layer [43].

The aptasensor was expected to behave in the following manner. Initially, the aptamer-modified electrode would be incubated with a chosen redox indicator that would result in a current response. Then, after electrode incubation with Pb^2+^ ions, the interaction between the target analyte and the sensing layer would cause the aptamer strands’ rearrangement and the formation of a G-quadruplex structure. Consequently, the formation of steric hindrance and the change in the overall negative charge of the sensing layer would lead to the alteration of the current response. To verify the possibility using the PS2M aptamer as a receptor element, four different redox indicators were tested: 1 μM methylene blue, 5 mM ferri/ferrocyanide redox couple, 100 μM AQMS, and 10 μM RuHex. It should also be noted that all the experiments were performed using Tris-HCl solution of pH 4.0 as for higher pH values, the hydroxide complexes of lead could be formed [42]. To choose the most appropriate electroactive indicator, the electrodes were firstly incubated solely with the redox active indicator for 5 min, which was followed by 5 min incubation with a redox active solution spiked with lead ions. The aptasensor response was set to be a relative current difference expressed as:

$$Aptasensor\;response=\frac{\left(I_{0}-I_{1}\right)}{I_{o}}$$

where I_0_ is the current before and I_1_ the current after electrode incubation with lead ions.

**Figure 1:**
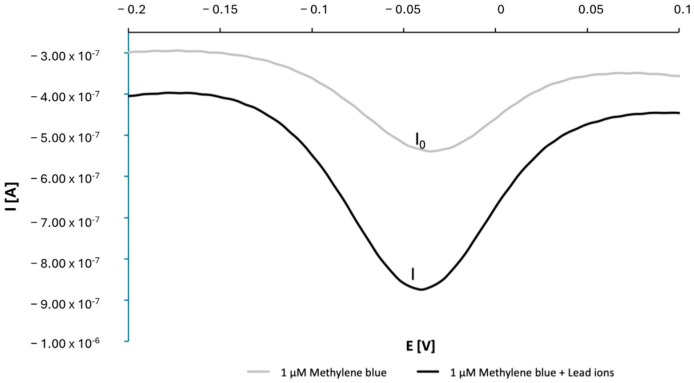
An exemplary square-wave voltammogram (cathodic scan) evidencing current increase originated from reduction in methylene blue molecules after incubation of aptamer-modified gold electrode in solution containing lead ions.

**Figure 2:**
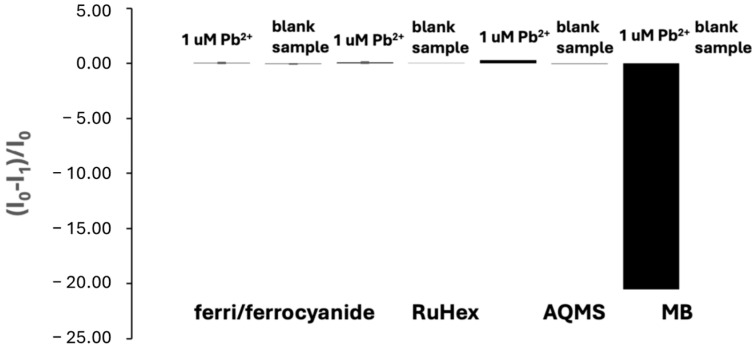
Comparison of aptasensor response after PS2M aptamer/MCH-modified electrode incubation in blank sample and solution of 1 μM lead ions in the presence of redox indicators: 5 mM ferri/ferrocyanide, 10 μM RuHex, 100 μM AQMS, and 1 μM methylene blue. All experiments were recorded using square-wave voltammetry (cathodic scan).

As it can be seen in Figure 1, the aptasensor response after electrode incubation with 1 μM lead nitrate was minor for all tested redox indicators expect for 1 μM methylene blue. In the case of ferri/ferrocyanide, AQMS, and RuHex, the aptasensor response was negligible both after electrode incubation with blank sample (solely redox indicator solution) and 1 μM lead nitrate solution. The reason for the distinct response when methylene blue was utilized could be its specific heterocyclic aromatic structure and the fact that it exhibits high affinity to guanine moieties [49]. Since the presence of lead ions causes the formation of a G-quadruplex structure, the guanines present in the aptamer strand become exposed to the outer part of the receptor layer and interact with MB molecules, resulting in an increase in current signal, which was observed in square-wave voltammograms (Figure 2). The possibility of MB interaction with nucleic acids is based on electrostatic attraction with negatively charged sugar-phosphate backbone as well as interaction between minor/major grooves and intercalation when secondary structures are formed. The advantage of the introduction of methylene blue concerns the occurrence of guanine nucleotides in PS2M for which MB exhibits high affinity [49]. This interaction is dominant in the designed aptamer-based layer and exceptional for methylene blue. Hence, no significant change in current response was observed when other redox indicators were added to the solution. It should also be noted that the use of methylene solution of a low concentration (1 μM) allows the researchers to detect current signal changes occurring in the receptor layer with negligible contribution of methylene blue molecules unbound to the aptamer-based layer [54]. Hence, the aptamer-based layer reorganization could be visualized as shown in Scheme 1.

Subsequently, after the confirmation of the possibility of using methylene blue as a redox indicator, the optimization of the aptamer-sensing layer content was performed that included the tuning of the aptamer and the blocking agent—6-mercapto-1-hexanol (MCH)—concentration. As can be seen in Figure S1, the highest current change after incubation with 1 μM lead ions was observed when 4 μM PS2M aptamer was applied, which could be justified by the fact that the maximum saturation of binding between Pb^2+^ ions and the aptamer layer among the tested aptamer concentrations was reached. The comparison of the influence of the MCH concentration on the aptasensor response revealed that a 4 μM concentration was sufficient to obtain the most intensive current response. Higher blocking agent concentration might have caused the removal of some of the aptamer strands from the receptor layer and led to a sensing layer of lower density (Figure S2). Therefore, both aptamer and MCH concentration were set to 4 μM.

**Figure 3:**
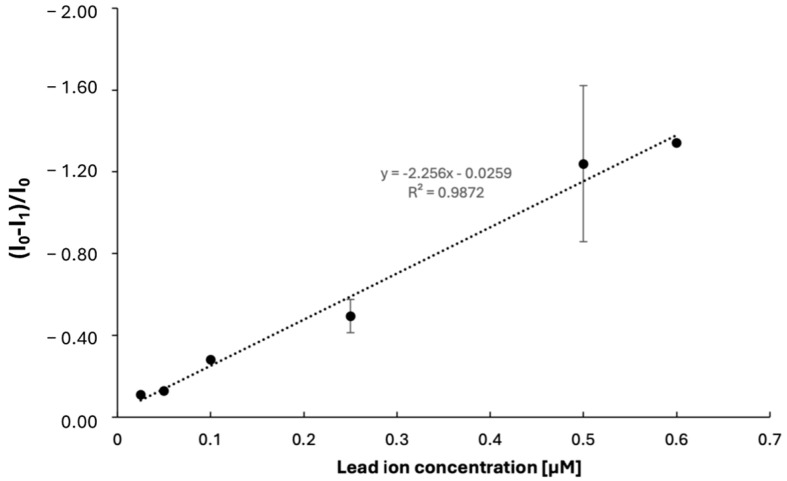
Calibration curve for PS2M-aptamer-based biosensor for the detection of lead ions. All the experiments were recorded using square-wave voltammetry (cathodic scan) in the presence of 1 μM methylene blue. The experiments were conducted in 3–4 repetitions. The black dots represent tested lead ions concentration, and a dotted line is a linear regression.

The next stage of research enabled the determination of the linear range of response towards lead ions. As can be seen in Figure 3, the aptasensor response changed linearly from 50 to 600 nM (with an LOD of 50 nM), which covers the range below the maximum allowed level of lead ions in water samples, that is 72 nM, set by US EPA [55], as well as allowing for the detection of a higher Pb^2+^ ion concentration. It can therefore be concluded that the developed aptasensor can be successfully applied to the determination of a wide range of lead ion concentrations.

**Figure 4:**
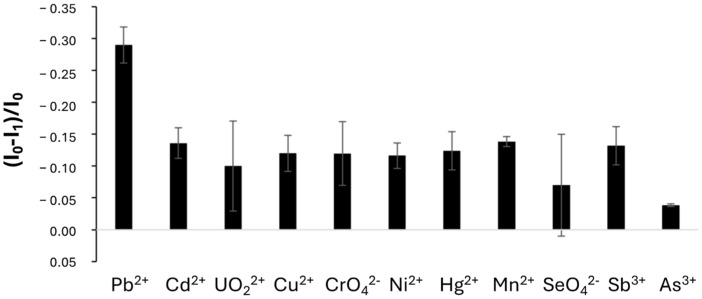
Selectivity studies for PS2M-aptamer-based biosensor. All the experiments were recorded using square-wave voltammetry (cathodic scan) in the presence of 1 μM methylene blue. The experiments were conducted in 3–4 repetitions.

**Figure 5:**
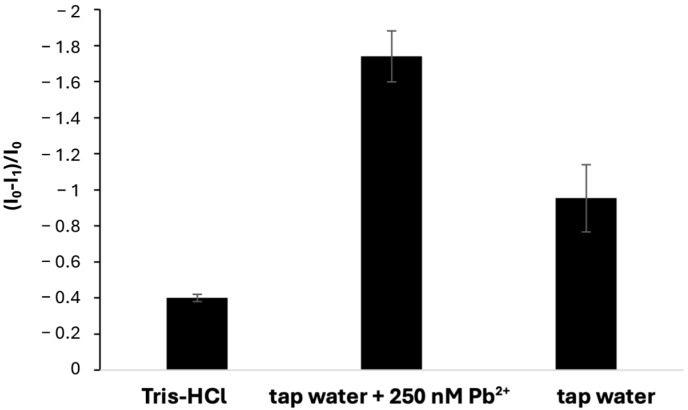
Comparison of aptamer-based biosensor response towards lead ions in a laboratory sample (Tris-HCl containing methylene blue) and a tap water sample diluted with Tris-HCl (containing methylene blue) without and spiked with 250 nM lead nitrate. All the experiments were recorded using square-wave voltammetry (cathodic scan) in the presence of 1 μM methylene blue. The experiments were conducted in 3–4 repetitions.

As the selectivity of elaborated aptamer-based layer is one its most important parameter, the response towards 72 nM Pb^2+^ ions and interfering ions at the same concentration were compared. It can be seen in Figure 4 that the (I_0_ − I_1_)/I_0_ for Pb^2+^ is at least two times higher than for other cations. Interestingly, all tested cations and anions caused a current increase, which could be explained by the aptamer-layer rearrangement and possibly the exposure of guanine moieties for which methylene blue shows high affinity. For interfering ions, the change in signal, which is at least 2 times smaller than for lead ions, might be explained by the partial accumulation of methylene blue molecules (which are present in all solutions that were spiked with interfering ions) during the 5 min incubation period; however, this did not cause the aptamer layer structural rearrangement and the agglomeration of MB indicator as observed for lead ions. Hence, the aptasensor response towards lead ions was substantial and points to the high selectivity of the PS2M aptamer/MCH layer towards Pb^2+^ ions. Further research was conducted with regard to the analysis of a real sample which was tap water spiked with 250 nM lead nitrate. For the comparison of the responses of the aptamer-based biosensor, a lead nitrate solution in 50 mM Tris-HCl and tap water diluted with Tris-HCl containing methylene blue without and spiked with lead ions were prepared. As can be seen in Figure 5, the response for the real sample spiked with lead ions was slightly higher than in the case of the laboratory sample, which can be explained by the presence of other components in tap water that could also cause a change in current response. Nevertheless, the utility of the proposed aptasensor was evidenced.

**Figure 6:**
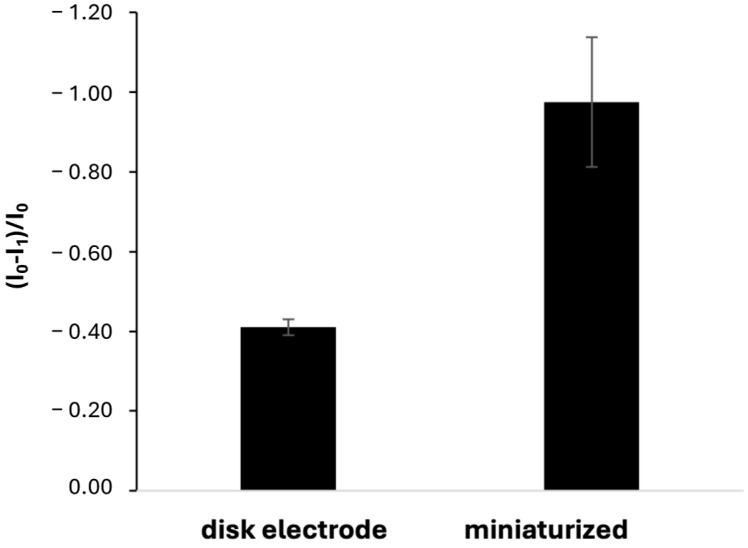
Comparison of aptamer-based biosensor response towards lead ions at 250 nM for gold disk electrodes and the miniaturized three-electrode system used as transducer elements. All the experiments were recorded using square-wave voltammetry (cathodic scan) in the presence of 1 μM methylene blue. The experiments were conducted in 3–4 repetitions.

The possibility of aptasensor miniaturization was also verified using screen-printed three-electrode chips. The aptamer-based layer was formed according to the same protocol as the working electrode modification, and was followed by electrochemical measurements identical to those applied to the classical three-electrode system. As can be seen in Figure 6, the aptasensor response for miniaturized electrodes was higher than for gold disk electrodes, and this could be explained by the possibly different aptamer-layer density for the two types of electrodes. Nevertheless, this gives an indication of an even better response of the proposed aptasensor fabricated on miniaturized chips.

**Figure 7:**
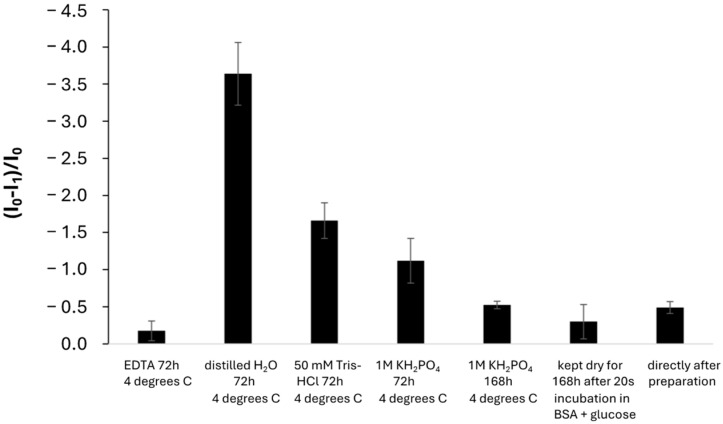
Comparison of aptamer-based biosensor responses towards lead ions at 250 nM for gold disk electrodes immediately after preparation and after storage in defined conditions. All the experiments were recorded using square-wave voltammetry (cathodic scan) in the presence of 1 μM methylene blue. The experiments were conducted in 3–4 repetitions.

**Figure 8:**
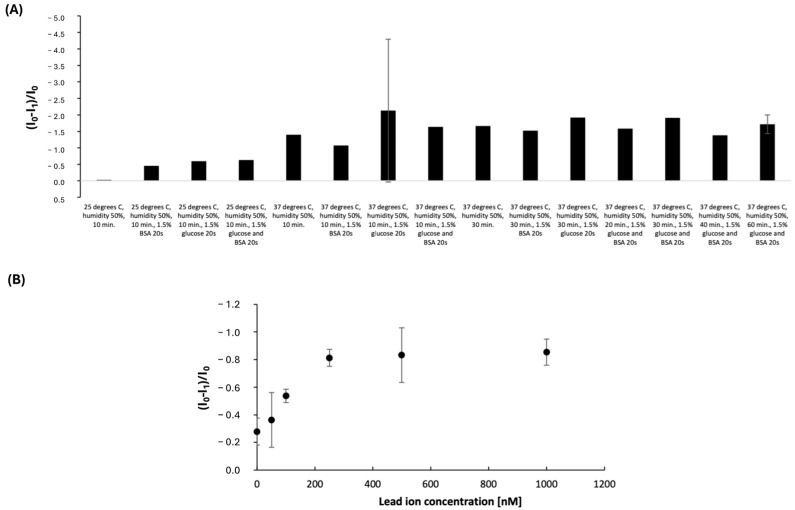
(**A**) Comparison of aptamer-based biosensor responses towards lead ions at 250 nM for gold disk electrodes immediately after preparation and after storage in a climate chamber. (**B**) Calibration curve for aptamer-based electrodes stored for 30 min at 50% humidity and 37 °C, preceded by 20 s incubation in 1.5% glucose and BSA solutions. All the experiments were recorded using square-wave voltammetry (cathodic scan) in the presence of 1 μM methylene blue. The experiments were conducted in 3–4 repetitions. The black dots in (**B**) represent defined concentrations of lead ions.

Concerning the future application of the elaborated aptasensor to lead ion detection, it was also vital to adjust the conditions for the storage of the aptamer-modified electrodes to achieve the detection of lead ions without a significant loss of signal. Firstly, we performed a comparison of the responses of aptamer-modified electrodes subjected to 250 nM Pb^2+^ ions directly after preparation and after 72 or 168 h storage at defined conditions. As can be seen in Figure 7, an exceptionally high response was evidenced after storage at 4 °C degrees in distilled water, followed by smaller but still high responses after electrode incubation in 50 mM Tris-HCl (pH 4.0) or 1M KH_2_PO_4_ (pH 4.5). In the first case, the significantly higher response of the biosensor might be caused by the partial removal of aptamer and blocking agent molecules from the gold surface. As a result, more methylene blue molecules could bind directly to the Au surface, thus contributing to the final current response. In the case of electrode incubation in either 50 mM Tris-HCl or 1M KH_2_PO_4_, the separation of some aptamer and 6-mercapto-1-hexanol molecules cannot be excluded, though to a smaller extent. To further verify the influence of temperature and humidity conditions on the aptamer-based layer response, we stored the electrodes in a climate chamber. The electrodes were subjected to storage between 10 and 60 min at 50% humidity at 25 and 37 °C. Additionally, prior to placing them in the climate chamber, in some cases, the electrodes were immersed in 1.5% BSA, 1.5% glucose, or 1.5% glucose and BSA in 1M KH_2_PO_4_. As can be seen in Figure 8A, the most significant responses were recorded after 10 min storage at 37 °C and 20 s incubation in 1.5% glucose, and after 30 min storage at 37 °C and 20 s incubation in 1.5% glucose or 1.5% glucose and BSA. Since a significant standard deviation was observed in the first condition, the latter two conditions appeared to provide the highest stability of the aptamer-based layer. Moreover, as the application of sugar and BSA as stabilizers has already been shown to reduce the biosensor response loss [56], 30 min storage at 37 °C and 20 s incubation in 1.5% glucose or 1.5% glucose and BSA were chosen as the final conditions for electrode storage. It can be seen in Figure 8B that a calibration curve showed a linear range of response from 0 to 250 nM of lead ions, which is far narrower than in the case of the aptamer-based biosensor used for analysis immediately after preparation. It should be emphasized that plot flattening is observed for higher concentrations, indicating the saturation of the aptamer layer with Pb^2+^ ions. Nevertheless, the PS2M aptamer/MCH-modified electrodes still allow for lead ion determination both below and above the maximum allowed Pb^2+^ level, which is 72 nM.

**Figure 9:**
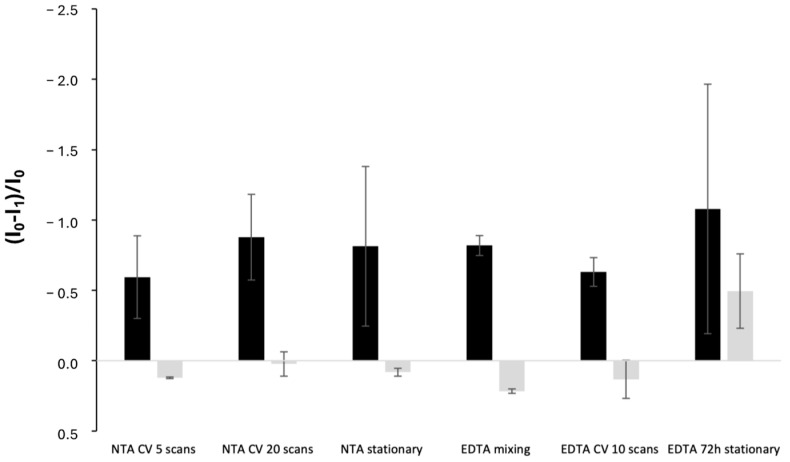
Comparison of aptamer-based biosensor responses towards lead ions at 250 nM for gold disk electrodes subjected to regeneration at defined conditions. All the experiments were recorded using square-wave voltammetry (cathodic scan) in the presence of 1 μM methylene blue. The experiments were conducted in 3–4 repetitions. The black columns represent aptasensor responses before regeneration whereas the grey ones after electrodes’ regeneration at defined conditions.

Finally, the possibility of PS2M aptamer-based layer regeneration was studied to reveal whether it is possible to apply the modified electrode more than once. For that purpose, after the first cycle of measurements in 250 nM lead ions, electrodes were immersed in solutions of 1 mM NTA or EDTA and kept for 5 min at stationary or stirring conditions as well as being subjected to electrochemical scanning using cyclic voltammetry in a range from −0.6 to 0.2 V. As can be seen in Figure 9, the only positive result for the second cycle of lead ion detection at a 250 nM concentration was obtained after 72 h electrode storage in 1 mM EDTA (in 50 mM Tris-HCl, pH 8.0) at 4 °C. The reason for that could be the partial damage of the sensing layer resulting from applying a defined potential range to the aptamer-modified electrodes and repetitive cycles of scanning, as well as solution agitation, which might result in the separation of some aptamer strands from the gold surface. Hence, 72 h storage in 1 mM EDTA at stationary conditions is the most promising procedure for aptamer-based layer regeneration and can allow for the electrodes’ further use in the next set of experiments aimed at Pb^2+^ ion detection.

## 4. Conclusions

The regular monitoring of lead ion concentration in water samples is vital as Pb can accumulate in living organisms and might lead to severe health problems or even death. This is the reason for the constant elaboration of novel tools for lead ion determination. In this study, we applied a PS2M aptamer that was previously applied to potassium ions using electrochemical methods or the optical detection of lead ions. The experiments involved the choice of a redox indicator that evidenced the binding between lead ions and the aptamer-based layer, and methylene blue was selected for electrochemical studies. This was followed by optimization studies on the content of the receptor layer including the concentrations of the aptamer and the MCH blocking agent. It was shown that the proposed aptasensor enabled lead ion detection from 50 to 600 nM, which covers the range below and above the maximum acceptable level of Pb^2+^ ions set by US EPA (72 nM). The developed aptasensor was shown to be selective towards lead ions as its response was at least 50% higher than that for other interfering ions at 72 nM concentration. The feasibility of usage of the proposed biosensor for the analysis of real samples was verified by the comparison of current responses for a laboratory sample and a tap water sample spiked with lead nitrate at 250 nM. Finally, the possibility of aptasensor miniaturization was checked by the application of screen-printed three-electrode chips, and the obtained response was more than two times higher than for classical gold disk electrodes. This gives an indication of an even better sensitivity of the aptasensor while using a miniaturized sensing system. It should also be noted that the storage of aptamer-based electrodes in a climate chamber for 30 min at 50% humidity and 37 °C, preceded by 20 s incubation in 1.5% glucose and BSA solutions, provided lead ion detection between 0 and 250 nM, with further saturation of the receptor layer with the target analyte. Finally, the PS2M aptamer layer could be regenerated by 72 h storage at 4 °C and used for another cycle of lead ion determination. The thorough studies on the application of the PS2M aptamer to the electrochemical detection of lead ions showed the advantage of applying the proposed aptasensor in terms of the limitation of operation time (see Table 1). Although the limit of detection is slightly smaller than in the case of a TBA-based aptasensor [42], it still allows for Pb^2+^ ion analysis below the maximum acceptable level, which is 72 nM [56].

Future studies could include the comparison of responses for other types of miniaturized electrodes, the analysis of various water samples, and the construction of a flow system that could allow for the automatization of the detection procedure.

## Figures and Tables

**Table 1:** Comparison of working parameters of aptamer-based sensors for detection of lead ions.

Receptor Layer	Detection Technique	LOD	Linear Range of Response	Operation Time	Reference
PS2M	Fluorescence	1 nM	5 nM–1 µM	30 min	[48]
PS2M	Colorimetry	2.5 nM	10–500 nM 0.5–25 nM	80 min	[49]
TBA/MCH	Square-wave voltammetry	34.7 nM	50–1000 nM	12 min	[42]
PS2M/MCH	Square-wave voltammetry	50 nM	50–600 nM	12 min	This work

## Data Availability

Data are contained within the article and Supplementary Materials.

## Supplementary Materials

The following supporting information can be downloaded at: www.mdpi.com/article/10.3390/bios15010059/s1, Figure S1: Comparison of aptasensor responses after incubation with 1 μM lead ions and blank sample using a sensing layer composed of either 1, 2, or 4 μM aptamer and 4 μM MCH. All the experiments were recorded using square-wave voltammetry (cathodic scan) in the presence of 1 μM methylene blue; Figure S2: Comparison of aptasensor responses after incubation with 1 μM lead ions and blank sample using a sensing layer composed of 4 μM aptamer and 4, 100 μM, or 2 mM MCH. All the experiments were recorded using square-wave voltammetry (cathodic scan) in the presence of 1 μM methylene blue.
