# Supplementary material for: Application of PS2M Aptamer as Receptor Layer for Electrochemical Detection of Lead Ions
Source: Biosensors (Basel). 2025 Jan 17;15(1):59. doi: 10.3390/bios15010059 (PMC11764081; doi:10.3390/bios15010059)
Supplement: Supplementary file 1 [file biosensors-15-00059-s001.zip › biosensors-3386331-supplementary.pdf]

Application of PS2M aptamer as receptor layer for electrochemical detection of lead ions

Izabela Zaras [1], Olga Kujawa [1], Marcin Olszewski [2] and Marta Jarczewska [1,*]

[1] Warsaw University of Technology, Faculty of Chemistry, Chair of Medical Biotechnology, Noakowskiego 3, 00-664 Warsaw, Poland
[2] Warsaw University of Technology, Faculty of Chemistry, Chair of Drug and Cosmetics Biotechnology, Koszykowa 75, 00-664 Warsaw, Poland
* Correspondence: marta.jarczewska@pw.edu.pl

Supplementary information

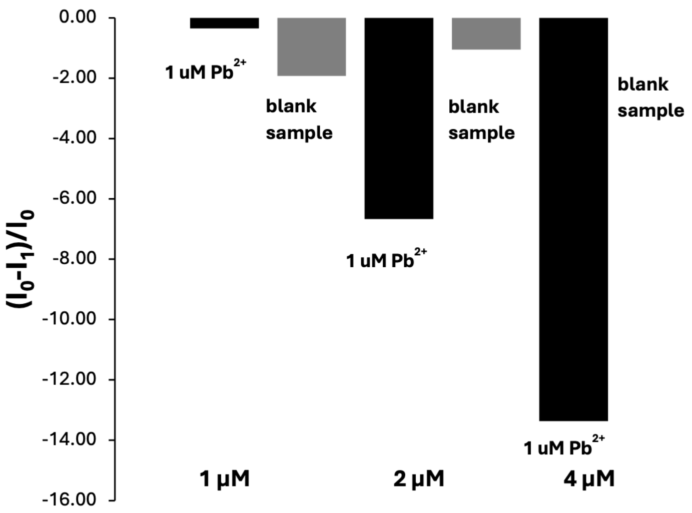

Figure S1. Comparison of aptasensor responses after incubation with 1 µM lead ions and blank sample applying sensing layer composed of either 1, 2 or 4 µM aptamer and 4 µM MCH. All the experiments were recorded using square-wave voltammetry (cathodic scan) in the presence of 1 µM methylene blue.

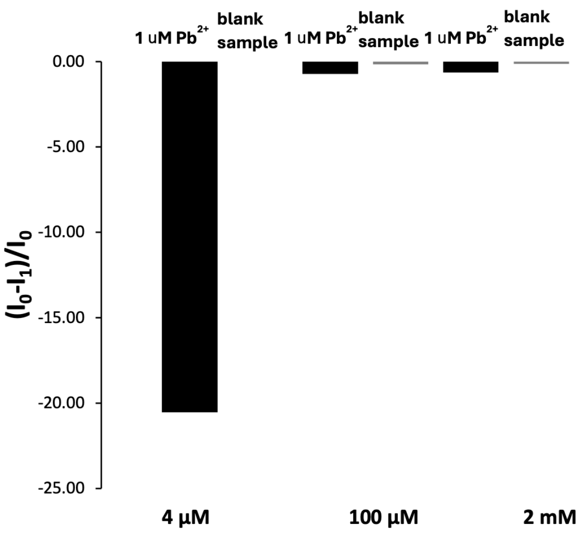

Figure S2. Comparison of aptasensor responses after incubation with 1 µM lead ions and blank sample applying sensing layer composed of 4 µM aptamer and 4, 100 µM or 2 mM MCH. All the experiments were recorded using square-wave voltammetry (cathodic scan) in the presence of 1 µM methylene blue.
